# Lyme Disease: A Role for Coenzyme Q10 Supplementation?

**DOI:** 10.3390/antiox11040667

**Published:** 2022-03-30

**Authors:** David Mantle, Nadia Turton, Iain P. Hargreaves

**Affiliations:** 1 Pharma Nord (UK) Ltd., Newcastle NE61 2DB, UK; dmantle@pharmanord.com; 2 School of Pharmacy and Biomolecular Sciences, Liverpool John Moores University, Liverpool L3 3AF, UK; n.m.turton@2020.ljmu.ac.uk

**Keywords:** Lyme disease, coenzyme Q10, fatigue, inflammation, oxidative stress

## Abstract

Lyme disease results from a bacterial infection following a bite from an infected tick. Patients are initially treated with antibiotics; however, in cases where antibiotic treatment is delayed, or when patients do not respond to antibiotic treatment, fatigue may develop alongside problems affecting the nervous system, cardiovascular system, and joints. It is thought that most of the damage to these tissues results from the excessive inflammatory response of the host, involving a self-reinforcing cycle of mitochondrial dysfunction, oxidative stress and inflammation. In this article, we review the potential role of supplementary coenzyme Q10 (CoQ10) in mediating the pathogenic mechanism underlying Lyme disease, on the basis of its role in mitochondrial function, as well as its anti-inflammatory and antioxidant actions.

## 1. Introduction

Lyme disease, first recognized in 1975, is an infection caused by spirochaete bacteria of the genus Borrelia, which is transmitted to humans following bites from infected ticks of the genus Ixodes [1]. Lyme disease is prevalent throughout the temperate Northern hemisphere, particularly in areas associated with woodland; the highest incidence is reported in central Europe, with approximately 200 cases per 100,000 population per year, and in Sweden with 600 cases per 100,000 population per year [2]. The precise incidence of Lyme disease in the UK is unknown, but has been estimated to be between 2000 and 10,000 cases per year [3]. Patients initially present with a characteristic circular skin rash at the site of the tick bite, and are typically treated with antibiotics such as doxycycline or amoxicillin [4]. In cases which are initially untreated or which do not respond to antibiotic treatment (variously estimated at 10–35%), problems affecting the nervous system, cardiovascular system, muscles, or joints may subsequently develop [5].

Following injection of the Borrelia bacteria into the skin, the bacteria multiply and spread to form the so-called erythema migrans lesion. These lesions, usually localized at the bite area, may become visible from a few days to up to 3 months after infection. The bacteria then access the blood circulation, where they continue to proliferate, to be distributed to tissues around the body, notably in the nervous system, heart, and joint tissues [5]. Although the bacteria have a variety of strategies to evade destruction by the innate or adaptive immune systems [6,7], it is thought that most of the damage to these tissues results from the inflammatory response of the host, involving a self-reinforcing cycle of oxidative stress and mitochondrial dysfunction [8]. In this article, we review the potential role of supplemental coenzyme Q10 (CoQ10), on the basis of its anti-inflammatory and antioxidant action, in mediating the pathogenic mechanism underlying Lyme disease.

## 2. Coenzyme Q10

CoQ10 is usually described as a vitamin-like substance which is, however, synthesized within most tissues in the human body. CoQ10 serves a number of key functions in cellular metabolism, most notably in the process of cellular energy supply via oxidative phosphorylation within the mitochondria, and also as a lipid soluble antioxidant protecting cellular and sub-cellular membranes from free-radical-induced oxidative damage [9]. CoQ10 also has a role in maintaining intra-lysosomal pH [10]. In addition, CoQ10 has been shown to directly mediate the expression of a considerable number of genes, including those involved in the process of inflammation [11]. There are currently more than 50 randomized controlled clinical studies relating to CoQ10 and inflammation in a variety of disorders listed on Medline, most of which have shown significant reductions in inflammatory markers following CoQ10 supplementation. Most of the body’s daily CoQ10 requirement (estimated to be approximately 500 mg, based on a body pool of 2000 mg and average tissue turnover time of 4 days) is derived from endogenous synthesis, although a small amount (approximately 5 mg) is obtained from the normal diet [12].

## 3. Lyme Disease, Fatigue, and CoQ10

Lyme disease patients may experience extreme fatigue, together with diminished cognitive function and musculoskeletal pain; this is particularly prevalent in patients with so-called post-treatment Lyme disease syndrome (PTLDS), in which such symptoms can persist for months following antibiotic treatment [13]. In this regard, patients with Lyme disease exhibit similar symptoms to those with chronic fatigue syndrome or fibromyalgia [14,15]. In such syndromes, fatigue may be associated with cellular energy depletion and mitochondrial dysfunction. To date, there have been no studies that directly measure possible host ATP depletion or defective oxidative phosphorylation in Lyme disease. However, evidence of more generalized mitochondrial dysfunction in Lyme disease was obtained in the study by Fitzgerald et al. [16]; in the metabolomics study by Fitzgerald et al. [16], a decrease in the serum level of the short- and medium-chain acylcarnitine species, acetylcarnitine and octanolycarnitine, respectively, was detected in patients with Lyme disease in comparison to healthy controls. This finding was interpreted as an indication of impaired mitochondrial function since the decreased level of acetylcarnitine may reflect a deficit in acetyl-CoA availability, which is the product of both the mitochondrial tricarboxylic acid cycle and fatty acid beta-oxidation [16]. However, no further investigation was made in the study by Fitzgerald et al. [16] which explored the cause of diminution in acetylcarnitine status or of generalized mitochondrial defects.

Mitochondrial dysfunction has been implicated in the pathogenesis of both chronic fatigue syndrome and fibromyalgia [17,18]. Randomized controlled clinical trials have demonstrated that supplementation with CoQ10 can significantly improve symptoms in both of these disorders [19,20]. More generally, a systematic review of interventional studies by Mehrabani et al. [21] identified the significant benefits of CoQ10 supplementation on fatigue following exercise, statin intake, and in patients with multiple sclerosis and end-stage heart failure. It therefore follows that supplementation with CoQ10 could potentially alleviate symptoms of fatigue in Lyme disease.

To date, only one preliminary study supplementing CoQ10 in Lyme disease has been reported in the medical literature. In a clinical study by Nicolson et al. [22], a series of 16 Lyme disease patients with persistent fatigue were supplemented with a combination of CoQ10, NADH (reduced nicotinamide adenine dinucleotide), and phosphoglycolipids. In addition to CoQ10, which would be expected to enhance residual mitochondrial function and enhance cellular antioxidant status, phosphoglycolipids were included to replace damaged cellular lipids and NADH was used as a substrate to enhance oxidative phosphorylation [22]. After 2 months, there was a significant 26% reduction in fatigue, as determined via the validated Piper Fatigue Scale. Analysis of subcategories of fatigue indicated that there were significant improvements in the ability to complete tasks and activities, as well as significant improvements in mood and cognitive abilities.

## 4. Lyme Disease, Inflammation, and CoQ10

Following initial entry into the skin, the B. burgdorferi infectious agent seems to possess the ability to induce persistent inflammation within the human host. This may be a result of the presence within the bacteria of strongly pro-inflammatory lipoproteins, which are capable of the induction of nuclear factor kappa beta (NFkb), and activating macrophages, neutrophils, mast cells, and lymphocytes, resulting in an excessive inflammatory response, ultimately with autoimmune characteristics [6]. It is possible that dead spirochetes—or fragments thereof—may persist and act as a focus for ongoing inflammation [23].

There is a common misconception that inflammation, which involves the release of pro-inflammatory cytokines, is a wholly negative process within the body. However, inflammation is the body’s normal response to infection or injury, and is essential for tissue healing, although this process should resolve itself following the initial immune response [24]. A balance must therefore be achieved in immune defense by neutralizing infectious organisms without precipitating their so-called cytokine storm—the uncontrolled release of pro-inflammatory cytokines responsible for tissue injury. The role of CoQ10 in mediating this process has been reviewed by Mantle et al. [25].

A number of randomized controlled clinical studies have reported significant reductions in various circulatory inflammatory markers (e.g., interleukins, tumor necrosis factor alpha, C-reactive protein) following supplementation with CoQ10 in a wide range of disorders; examples include cardiovascular disease [26], non-alcoholic fatty liver disease [27], and polycystic ovary syndrome [28]. It follows that supplementary CoQ10 may be effective in reducing inappropriate inflammation in Lyme disease, thereby mediating subsequent tissue damage.

## 5. Lyme Disease, Oxidative Stress, and CoQ10

Evidence for a role of oxidative stress in the pathogenesis of Lyme disease has been obtained from several clinical studies (performed on patients who had not been treated with antibiotics). In a study by Peacock et al. [29], mitochondrial superoxide levels were found to be significantly higher in blood mononuclear cells from a series of 32 Lyme disease patients, compared to controls; in addition, cytosolic calcium levels (a marker for disrupted intracellular communication and host response to infection) were significantly lower in Lyme disease patients compared to controls. The authors concluded that these results indicate an imbalance of reactive oxygen species and cytosolic calcium in Lyme borreliosis patients. The results further suggest that oxidative stress and interrupted intracellular communication may ultimately contribute to a condition of mitochondrial dysfunction in the immune cells of Lyme borreliosis patients.

In a separate study by Luczaj et al. [30], a series of 19 patients with Lyme disease arthritis were found to have significantly increased levels of lipid peroxidation products (malondialdehyde, 4-hydroxynonenal) in plasma and urine, compared to controls. Similarly, in a study by Moniuszko-Malinowska et al. [31], a series of 22 patients with the neurological manifestation of Lyme disease (neuroborreliosis) were found to have significantly increased levels of lipid peroxidation markers (4-hydroxynonenal, 4-hydroxyhexenal, malondialdehyde,4-oxononenal, F2-isoprostanes, and A4/J4-neuroprostanes) in CSF (cerebrospinal fluid), plasma, and urine, compared to controls. In a series of 20 Lyme disease patients with erythema migrans, plasma levels of antioxidant enzymes (superoxide dismutase, glutathione reductase, glutathione peroxidase), together with reduced glutathione, were significantly reduced compared to controls, while lipid peroxidation (quantified as malondialdehyde) was significantly increased. Finally, a comparison of the serum proteome of Lyme disease patients and controls revealed increased oxidative stress in the former [32]. Data from the above studies indicate a role for increased oxidative stress in various tissues in both early and later stages of Lyme disease pathogenesis. It is of note that the determination of the above markers of oxidative stress in plasma, urine, or CSF, particularly in the early stages of Lyme disease, has been used to monitor the course of treatment with antibiotics [33].

More than 100 randomized controlled clinical trials have demonstrated significant benefit of supplementation with CoQ10 on oxidative stress parameters in a wide range of disorders, including cardiovascular disease [34], multiple sclerosis [35], non-alcoholic fatty liver disease [36], and type II diabetes [37]. Given that supplementary CoQ10 is capable of significantly reducing oxidative stress in such a diverse range of disorders, one can predict that supplementary CoQ10 should also be capable of reducing oxidative stress in Lyme disease, thereby mediating the pathogenic mechanism.

## 6. Lyme Disease, Cardiovascular Dysfunction, and CoQ10

Lyme carditis occurs in up to 10% of Lyme disease patients, typically those with atrioventricular heart block, which may require temporary pacing; however, some patients may go on to develop heart failure [38]. An adequate supply of CoQ10 is of particular importance in tissues with a high-energy requirement such as the heart, and deficiency of CoQ10 has been implicated in the development of heart failure. In a randomized controlled clinical trial, supplementation with CoQ10 significantly improved symptoms in patients with heart failure, and reduced the risk of mortality by approximately 50% [39]. We therefore hypothesize that supplementation with CoQ10 may support normal cardiac function in Lyme disease patients, and help to mediate symptoms in those patients at risk of developing heart failure.

## 7. CoQ10 Supplementation: Safety and Importance of Formulation Type

The safety of CoQ10 has been confirmed in more than 200 randomized controlled clinical studies reported in the peer-reviewed medical literature, as listed on Medline. In these studies, CoQ10 has been supplemented in a wide range of disorders with no serious adverse events reported; very rarely, individuals may experience mild gastrointestinal disturbance. There are no known toxic effects, and CoQ10 cannot be overdosed [40]. Because of the structural similarity between CoQ10 and vitamin K, it has been suggested that CoQ10 could interfere with the anti-coagulant action of warfarin. However, a randomized controlled clinical study reported that supplementation with CoQ10 (100 mg/day) had no significant effect on the action of warfarin. On this basis, in general terms, it should be possible to take CoQ10 and warfarin together, although international normalized ratio (INR) values (used to determine the effects of anticoagulants on blood clotting) should be monitored in individual patients. An alternative strategy is to use anti-coagulants, such as rivaroxaban or apixaban, which act via a different mechanism to warfarin and do not have the same potential interaction with CoQ10. The variation in bioavailability between different CoQ10 supplements has been demonstrated in a double-blind crossover clinical study by Lopez-Lluch et al. [41]. Seven supplements, each containing 100 mg of CoQ10 but otherwise differing in formulation, were evaluated in a series of fourteen young healthy subjects. Bioavailability was quantified as the area under the curve of plasma CoQ10 levels over 48 h after ingestion of a single dose. Each supplement was investigated in turn within the same group of volunteers, with a minimum wash-out period of four weeks between each sample. There were statistically significant differences in the bioavailability of the CoQ10 supplements, with the highest bioavailability shown by a softy-gel capsule, in which the CoQ10 was subject to a patented crystal dispersion process, the absence of which reduced bioavailability by 75%. It is of note that the latter formulation received a marketing authorization within the EC, demonstrating the importance of utilizing a CoQ10 product manufactured to pharmaceutical standards, rather than food supplement standards.

## 8. Monitoring of CoQ10 Status in Lyme Disease Patients

In view of the evidence of oxidative stress and chronic inflammation, together with the implication of mitochondrial dysfunction associated with Lyme disease [6,16,31], the possibility arises that patients with this condition may have some underlying CoQ10 deficiency associated with the disease pathophysiology which has yet to elucidated. Clinical monitoring of CoQ10 status generally involves plasma determinations, with an established reference interval ranging from 0.5 to 1.7 μM [42]. Considering that plasma CoQ10 status is influenced by both diet and circulatory lipoprotein levels, the latter being the major carriers of CoQ10 in the circulation, it may be an inappropriate surrogate for assessing endogenous CoQ10 status [43]. For this reason, blood mononuclear cells or urine epithelial cells have been suggested as appropriate alternative surrogates for this determination [43]. The “gold standard” for the assessment of endogenous CoQ10 status is by skeletal muscle biopsy, although this is a relatively invasive procedure. Currently, there is no consensus on the appropriate dosage of CoQ10 or the plasma level required to engender an improvement in MRC (mitochondrial respiratory chain) function and/or to ameliorate oxidative stress or chronic inflammation. In vitro cell studies have indicated that a target plasma level of 5 µM may have the propensity to ameliorate mitochondrial dysfunction and decrease oxidative stress [44]. This target level of CoQ10 is further supported in a Parkinson disease study where a plasma CoQ10 level of 4.6 μmol/L was reported to be the most effective in slowing functional decline. However, this beneficial effect was thought to be the result of the antioxidant and mitochondrial functioning properties of CoQ10, rather than of its effect on the immune system [45]. Unfortunately, no studies have yet assessed the plasma CoQ10 status of Lyme disease patients, where evidence of clinical benefit has been noted following CoQ10 supplementation.

## 9. Conclusions

**Figure 1:**
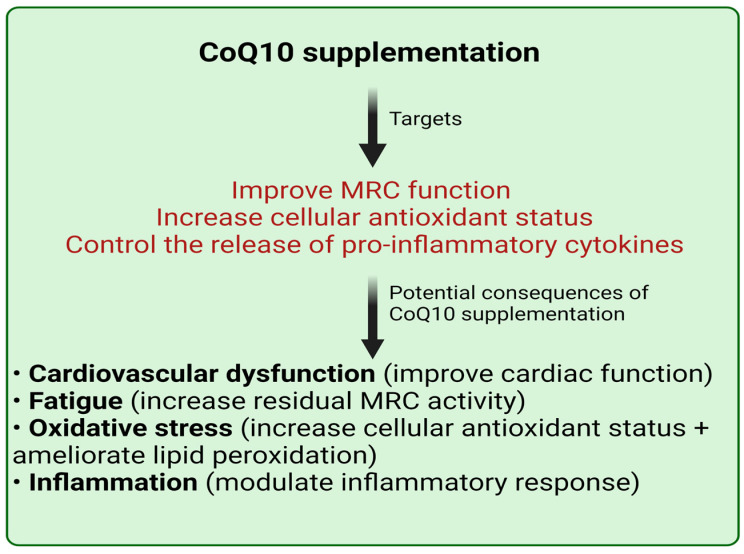
The potential targets and consequences of CoQ10 supplementation in Lyme disease. CoQ10: coenzyme Q10; MRC: mitochondrial respiratory chain.

CoQ10 is of relevance to Lyme disease on the basis of (i) its key role in mitochondrial energy supply, thereby helping to prevent mitochondrial dysfunction, impaired cellular energy supply, and subsequent fatigue; (ii) its role as an antioxidant, helping to counter the effects of free radical induced oxidative stress; (iii) its role as a mediator of the inflammatory process, helping to counter the tissue damaging effects of dysregulated inflammation; and (iv) its role in countering abnormal heart function. The potential therapeutic value that CoQ10 supplementation may have in Lyme disease is outlined in Figure 1.
